# The CRM1-dependent NES^257−266^ motif in the matrix protein: another factor influencing Newcastle disease virus propagation and virulence

**DOI:** 10.1186/s13567-025-01552-6

**Published:** 2025-07-01

**Authors:** Yaodong Zhang, Jun Dai, Lei Tan, Daoe Mu, Ziyan Zhang, Cuiping Song, Yang Qu, Ning Tang, Ying Liao, Chan Ding, Yingjie Sun, Xusheng Qiu

**Affiliations:** 1 Shanghai Veterinary Research Institute, Chinese Academy of Agricultural Sciences, Shanghai, 200241 China; 2 Experimental Animal Center, Zunyi Medical University, Zunyi, 563000 China; 3 Jiangsu Co-Innovation Center for Prevention and Control of Important Animal Infectious Diseases and Zoonoses, Yangzhou University, Yangzhou, 225009 China

**Keywords:** Newcastle disease virus, M protein, NES, CRM1, replication, virulence

## Abstract

As an important structural protein of Newcastle disease virus (NDV), the M protein plays a crucial role in the assembly and budding of the virus. In previous reports, we confirmed the existence of a classical nuclear export signal (NES) sequence on the M protein (i.e*.*, amino acids 257 to 266), which plays an important role in the nuclear export of the protein. In this study, we found that the NES^257−266^ motif is associated with the evolutionary history of NDV, and found that the NES-mediated nuclear export efficiency of M proteins varies among different genotypes. Further research on the LaSota-NES and JSD0812-NES variants showed that the NES both affects the nuclear-cytoplasmic trafficking efficiency of M protein and influences the formation of virus-like particles (VLPs). The NES^257−266^ conforms to the chromosome region maintenance 1 (CRM1)-associated consensus leucine/isoleucine-rich NES motif, and our results confirm that the NES-mediated nuclear-cytoplasmic trafficking of M protein is dependent on the CRM1 pathway. At last, several recombinant LaSota strains were rescued using reverse genetics, with their NES sequence replaced by either a potent NES motif, or an R247K mutation, or both. The rescued rLaSota-QMS and rLaSota-FM strains showed elevated hemagglutination (HA) titers, increased 50% egg infective dose (EID_50_) values, along with marginally higher mean death time (MDT) and intracerebral pathogenicity index (ICPI) values, all of which are attributable to their enhanced proliferation rates when compared to the wild-type (wt) rLaSota strain. Our findings contribute to a better understanding of the molecular mechanisms of replication and pathogenicity in NDV and, by extension, in other paramyxoviruses.

**Supplementary Information:**

The online version contains supplementary material available at 10.1186/s13567-025-01552-6.

## Introduction

Viruses of the genus *Avian orthoavulavirus 1* (AOAV-1), commonly known as Avian paramyxoviruses 1 (APMV-1) or Newcastle disease viruses (NDV), cause infection in a wide range of domestic and wild birds worldwide [1, 2]. Strains of NDV are phylogenetically classified into two classes, designated as Class I and Class II, and further subdivided into several genotypes based on their genetic differences. Class I viruses are genetically less diverse, generally present in wild waterfowl, and of low virulence [3]. Compared to class I, class II viruses are genetically and phenotypically more diverse, including most of the virulent NDV isolates and some of the non-virulent isolates, and have been classified into at least 20 distinct genotypes (I to XXI) [4]. Genotypes III to IX viruses represent highly virulence, while genotypes I and II are predominately viruses of low virulence [5]. Both of the coding sequence of the fusion gene and the entire genomes of viruses from wild birds displayed higher annual rates of change in virulent viruses than in viruses of low virulence, suggesting that increased virulence may accelerate the rate of NDV evolution. The early lineage included five genotypes (I to IV and IX) while the recent lineage consisted of four genotypes (V to VIII) [6]. With the evolution of the viruses and the advancement of genotyping methodologies, more genotypes, including XI-XXI, have been identified in recent years [2, 4]. For example, genotype XII viruses were isolated in sick chickens in South America and geese in China between 2008 and 2011 [7, 8], and genotype XXI viruses were identified from chickens and pigeons in different Asian, European, and African countries between 2005 and 2016 [9–12]. Genotype XIII strains previously classified as genotype VII were identified between 1982 and 2013 [13, 14]. Similarly, genotype XV comprises viruses obtained from chickens and geese in China, which have been previously classified into sub-genotype VIId or VIIe [15]. Genotype XIX contains all viruses previously classified as sub-genotype Va [16, 17]. Certain viruses previously classified as the sub-genotypes belonging to genotype VI were re-classified into two new genotypes, namely XX and XXI [4].

The NDV genome is an unsegmented single-negative-stranded RNA, about 15 kb, encoding six structural proteins, namely nucleoprotein (NP), phosphoprotein (P), matrix (M) protein, fusion protein (F), hemagglutinin-neuraminidase (HN) and large protein (L) [18]. NDV M protein serves as the bridge between NP and virus envelope, plays a central role in the process of viral budding and assembly, and is also involved in the escape of innate immunity [19–21]. Furthermore, M protein, as an important structural protein in virion morphogenesis, interacts with host cell proteins through its own special amino acid motif or site to promote viral proliferation [22–24]. Like most paramyxoviruses, although the whole replication cycle of NDV occurs in the cytoplasm, despite this, the M protein is found to be a nuclear-cytoplasmic trafficking protein during NDV infection [25]. This nuclear-cytoplasmic localization feature of NDV M protein is demonstrated to inhibit host cell transcription, regulate viral RNA synthesis and transcription, which promotes viral replication [26]. Moreover, mutations at sites 247 and 263 of M protein have been found to change the nuclear shuttle efficiency of M protein, thereby improving the viral replication ability and pathogenicity [23].

The molecular mechanism underlying the nuclear shuttling of the NDV M protein is still poorly understood. It is widely recognized that the majority of cargo proteins require energy-dependent transport to traverse the nuclear pore complex (NPC) for either nuclear entry or exit [27]. This transport is facilitated by cellular receptors that recognize and bind to specific short amino acid sequences—nuclear localization signals (NLS) for nuclear entry and nuclear export signals (NES) for exit [28]. The nuclear import of the M protein is commonly associated with a biphasic effect, which includes the inhibition of the transcription and translation of host proteins, as well as facilitating viral replication and virion budding [26].

Transport signals are recognized by karyopherin β (Kap β, also know as importin and exportin) family, which shuttle between the nucleus and cytoplasm [29, 30]. It has been reported that the NLS of the NDV M protein binds to the RanGTP-binding domain of IMPβ1 in the cytoplasm and then crosses the NPC into the nucleus with the help of RanGTP [31]. A typical NES sequence is characterized by ΦX(2–3)ΦX(2–3)ΦXΦ, where Φ refers to Lys, Val, Ile, Phe, or Met, X denotes any amino acid, and the number in parentheses indicates the number of repeats [32]. NES sequences matching this feature found in various cellular and viral proteins are leucine-rich [33]. Chromosome region maintenance 1 (CRM1), also known as exportin-1, is a classic nuclear transport receptor and responsible for the nuclear export of hundreds of proteins through the recognition of the leucine-rich NES [34]. The large hydrophobic residues are the most conserved in NES motifs which suggests that the interaction with CRM1 is mediated through these hydrophobic residues [32].

Previous studies have confirmed that the interaction between NES and CRM1 is pivotal in the nuclear export of the M protein in paramyxoviruses [35, 36]. Nonetheless, the exact role of the CRM1-mediated nuclear export pathway during NDV infection has yet to be definitively elucidated [37]. Duan et al. have identified three NES motifs within the M protein, designated as NES^169−178^, NES^235−245^, and NES^314−327^. These motifs exhibit high conservation across various genotypes, and bioinformatics analysis suggests they conform to the CRM1-associated consensus leucine/isoleucine-rich NES motif. Nonetheless, subsequent studies have shown that the nuclear export of the M protein through these motifs is independent of CRM1, and the influence of these motifs on NDV remains not fully understood.

In our recent study, a novel NES motif in the NDV M protein was identified and verified, although it varies among different genotypes [23]. Our research has elucidated that NES proteins play a role in modulating viral replication and virulence by impacting the nuclear export efficiency of M protein, thus the differences in NES between different genotypes suggest a new perspective for the study of the evolution of NDV virulence. In this study, we delved into the influence of NES motifs within the M protein from various NDV strains, which are representative of different eras, on the nuclear export efficiency of the M protein, as well as the virulence evolutionary patterns involved. Our findings have established a robust foundation for unraveling the nuclear transport mechanisms of M proteins in paramyxoviruses.

## Materials and methods

### Cells, viruses, and viral infection

Chicken embryo fibroblast (DF-1), human embryonic kidney 293 T (293 T), and HeLa cells were purchased from the American Type Culture Collection (ATCC) and cultured in Dulbecco’s modified Eagle’s medium (DMEM; Gibco, Grand Island, NY, USA) containing 10% fetal bovine serum (FBS; ExcellBio) at 37 ℃ and 5% CO_2_. The NDV strain LaSota/46 was purchased from the China Institute of Veterinary Drug Control (Beijing, China). Recombinant rLaSota-wt, rLaSota-R247K strains were rescued and preserved in our laboratory [23]. All viruses were propagated in 9 to 11-day-old specific-pathogen-free (SPF) embryonated chicken eggs. Allantoic fluid of SPF chicken embryos was collected at 24 h to 120 h after virus inoculation, and then the fresh allantoic fluid was collected and stored at −80 ℃. DF-1 and HeLa cells were infected with NDV at an multiplicity of infection (MOI) of 5 and incubated at 37 °C. For infection, DF-1 and HeLa cells were inoculated with NDV at a MOI of 5 in a 37 ℃ cell-culture incubator. After incubation in serum-free DMEM medium for 2 h, the cells were washed three times with phosphate-buffered saline (PBS) to remove unattached virus particles. Subsequently, the medium was replaced with DMEM containing 2% FBS, and samples were collected at 24 to 72 hours post-infection (hpi).

### Antibodies and chemicals

Anti-mouse-Flag (M185-3L) was purchased from MBL (Nagoya, Japan). Anti-rabbit-Flag (14793S) was purchased from Cell Signaling Technology (Beverly, MA, USA). Anti-β-actin (AC026) was purchased from Abclonal (Wuhan, China). Lamin B1 Antibody (MA9196S) and Beta-tubulin Antibody (MA8281S) were purchased from Abmart (Shanghai, China). Goat Anti-Rabbit IgG (H + L) (111–035-003) conjugated and goat anti-mouse IgG (H + L) (115–035-003) with HRP were from Jackson ImmunoResearch (West Grove, PA, USA). Alexa Fluor goat anti-rabbit-488 (A-11034), Alexa Fluor goat anti-mouse-488 (A-11029), were obtained from Invitrogen. The nuclear export inhibitor Leptomycin B (S1726; Beyotime) was used at a concentration of 10 ng/mL. The proteasome inhibitor MG-132 (HY-13259; MedChemExpress) was used at a concentration of 10 μM.

### Plasmids and mutagenesis

The NDV-M protein eukaryotic expression plasmids pCMV-14-LaSota-M-3 × FLAG, pCMV-14-Herts/33-M-3 × FLAG, pCMV-14-JSD0812-M-3 × FLAG, and the reporter plasmids Rev(1.4)-GFP (Vector) were constructed according to previous reports [23]. For reverse genetics, the full-length cDNA clones of LaSota-C5 and pLaSota and three helper plasmids, namely, pCI-NP, pCI-P, and pCI-L, had been previously constructed in our lab [23, 38]. All mutations, deletions, and insertions introduced into the expression plasmids, reporter plasmids, or NDV cDNA clones were performed using Mut Express II Fast Mutagenesis Kit V2 (Vazyme Biotech Co., Ltd., China). All the recombinant plasmids constructed in this study were listed in Additional file 2, and the sequences of all the primer used in this study are available upon request.

### Immunofluorescence

HeLa cells were cultured on a six-well plate and transfected. After transfection, the coverslips were washed with PBS, fixed with 4% neutral formaldehyde at room temperature for 30 min, then permeabilized in phosphate-buffered saline with Tween 20 (TBST) containing 0.5% Triton X-100 for 10 min, washed three times with 1 × TBST and blocked with 3% bovine serum albumin at 37 ℃ for 1 h. Then, cells were incubated with the primary antibody at 37 °C for 2 h. Cells were washed three times with 1 × TBST and incubated with the secondary antibody.

Subsequently, the cells were washed again and incubated with 0.5 mg/mL 4′,6-diamidino-2-phenylindole (DAPI). After washing again, the slides were fixed with a sealer and then observed with a Zeiss LSM 880 confocal microscope (Carl Zeiss, Jena, Germany).

ImageJ software was used to detect the fluorescence intensity of M proteins in the nucleus and cytoplasm. Briefly, 3–5 areas of each image were selected at random. The ratio of nuclear/cytoplasmic fluorescence intensity of the cells in the selected region was then calculated. About 30 cells were counted and analyzed for each image.

### Western blot (WB) analysis

Cell samples were washed with PBS and lysed with 2 × SDS loading buffer (2% sodium dodecylsulfate [SDS], 10% glycerin, 5% 2-mercaptoethanol and 0.1% bromophenol blue). Denaturation was performed at 100 °C for 10 min. An equal amount of these prepared samples was subjected to SDS–polyacrylamide gel electrophoresis and then transferred to nitrocellulose membranes (Whatman International, Ltd.). The membranes were then blocked with 5% non-fat milk in 0.05% TBST for 1 h at room temperature. The membranes were washed thrice with 0.05% TBST (5 min/each) and inoculated with primary antibodies for at least 8 h at 4 °C. The membranes were washed thrice again with 0.05% TBST (5 min/each), and incubated with HRP-conjugated secondary antibodies at room temperature for 2 h. Lastly, after washing thrice again, the antibody–antigen complex was exposed with a chemiluminescence reagent solution kit (Share-bio Biotechnology, Shanghai, China) using a multi-chemiluminescence image analysis system (Tanon 5200, Tanon, Guangzhou, China) and quantified using Image J software.

### Production and detection of viral-like particles (VLPs)

HEK-293 T cells were cultured in six-well plates, and each well was transfected with 2 μg plasmid expressing FLAG-M or M protein mutants. 24 and 48 h after transfection, 6 mL of culture supernatant was collected and centrifuged at 5000 × *g* for 10 min to remove cellular debris. The clarified supernatant was loaded into the filtration unit of the Amicon Ultra 100 K device (Merck) and centrifuged at 3000 ×*g* for 10 min. The concentrated supernatants were harvested and supplemented to 100 μL for each sample with PBS. The supernatant is then mixed with 5 × SDS-PAGE loading buffer. Meanwhile, at the same time point, the cell samples were washed with PBS and split with 2 × SDS loading buffer. Subsequently, the collected supernatant and cell samples were incubated at 100 °C for 10 min, and then used for Western blot (WB) assay.

### Sequence alignment and structure modeling

The nucleotide sequences of M and F proteins of different NDV strains were obtained from GenBank. Clustal W of MegAlign software was used to analyze the similarity of nucleotide sequences of M protein and F protein. The Neighbor-Joining method of MEGA11 software was used to construct the genetic evolutionary tree, and the self-spreading value was set to 1000. The structural models of LaSota-M proteins were constructed based on the former crystal structure of the NDV-M protein (PDB: 4G1G). All structural annotations were generated using PyMOL (version 3.1; Schrödinger).

### Recombinant virus recovery

The recovery of infectious recombinant LaSota strains followed as previously described in published articles in our laboratory [23]. Briefly, 2 μg pCI-NP; 2 μg pCI-P; 1 μg pCI-L and 5 μg pLaSota-QMS/FM plasmids were co-transfected into BSR T7/5 cells with Lipofectamine 2000 (Invitrogen). The transfected cells were cultured with 1% FBS and 2% pancreatic enzyme at 37 ℃ and 5% CO_2_. At 60 hours post-transfection (hpt), supernatant and monolayer cells were collected, mixed and inoculated into the allantoic cavity of 9-day-old SPF embryonated chicken eggs. Allantoic fluid was collected 4 days after inoculation and measured by hemagglutination (HA) titer test [39]. The rescued virus was then passed through successive generations (up to 5 times) in SPF embryonated eggs for further study.

### Biological characterization of the generated virus and in vitro growth kinetics

The standard pathogenicity tests for NDV, involving mean death time (MDT) and intracerebral pathogenicity index (ICPI), were performed to ascertain the differences in virulence between recombinant viruses, including rLaSota-wt, -R247K, -QMS and -FM, according to the instructions of the World Organization for Animal Health (WOAH) [40, 41].

To compare the influence of mutations on viral multiplication capacity, growth curves were generated in DF1 cells and Hela cells for rLaSota-R247K, -QMS, -FM and rLaSota-wt. All cells were cultured in 6-well plates (1 × 10^6^ cells/well) in DMEM containing 10% FBS and then infected with recombinant virus at a MOI of 5. At 6, 12, 18, 24, 30, and 36 hpi, the supernatants were harvested for virus titration via real-time reverse transcriptase PCR (RT-qPCR) or a 50% tissue culture infective dose (TCID_50_) test. For TCID_50_ assay, briefly, BHK-21 cells were seeded in 96-well plates and infected with serial tenfold dilutions (100 μL) of the supernatant, in 8 replicates. After incubation at 37 °C for 30 min, the supernatant was washed away and DMEM containing with 1% FBS and 2% pancreatic enzyme was added. Infected cells were cultured at 37 °C and 5% CO_2_ and observed for 96 h. Following the Reed-Muench method, virus titers were calculated by determining the dilution that yielded 50% of cells displaying cytopathic effects [42].

### Real-time RT-PCR quantification of viral RNA copies

Total viral RNAs were extracted from the supernatant of recombinant NDV (rNDV)-infected cells with Trizol reagent (Ambion) at different time points [43], and then reverse transcribed into cDNA by EasyScript^®^ One-Step gDNA Removal and cDNA Synthesis SuperMix (TransGen Biotech Co., Ltd., China) kit. Bio-Rad CFX-96 real-time fluorescence quantitative PCR instrument and SYBR green masterbatch (Guangzhou Dongsheng Biotech Co., Ltd., China) were used for quantitative PCR using cDNA as template. PCR conditions were as follows: an initial denaturation at 94 °C for 3 min, 40 cycles of 94 °C for 15 s, 60 °C for 15 s and 72 °C for 20 s. The specificity of the qPCR primers was monitored with melting curve analysis. In this study, real-time fluorescent quantitative PCR primers for determining virus copy number were used as in previous studies [23].

### Statistical analysis

The nuclear-cytoplasmic localization of M proteins, reporter genes, and viral HA titers were statistically analyzed, and *P* values were generated by analysis of variance (ANOVA) with Bonferroni correction for multiple comparisons between groups. All graphs and statistical analyses were generated with Prism 8 (GraphPad Software).

## Results

### The amino acid sequences of M protein’s NES motif are linked to the evolution history of NDV

**Figure 1:**
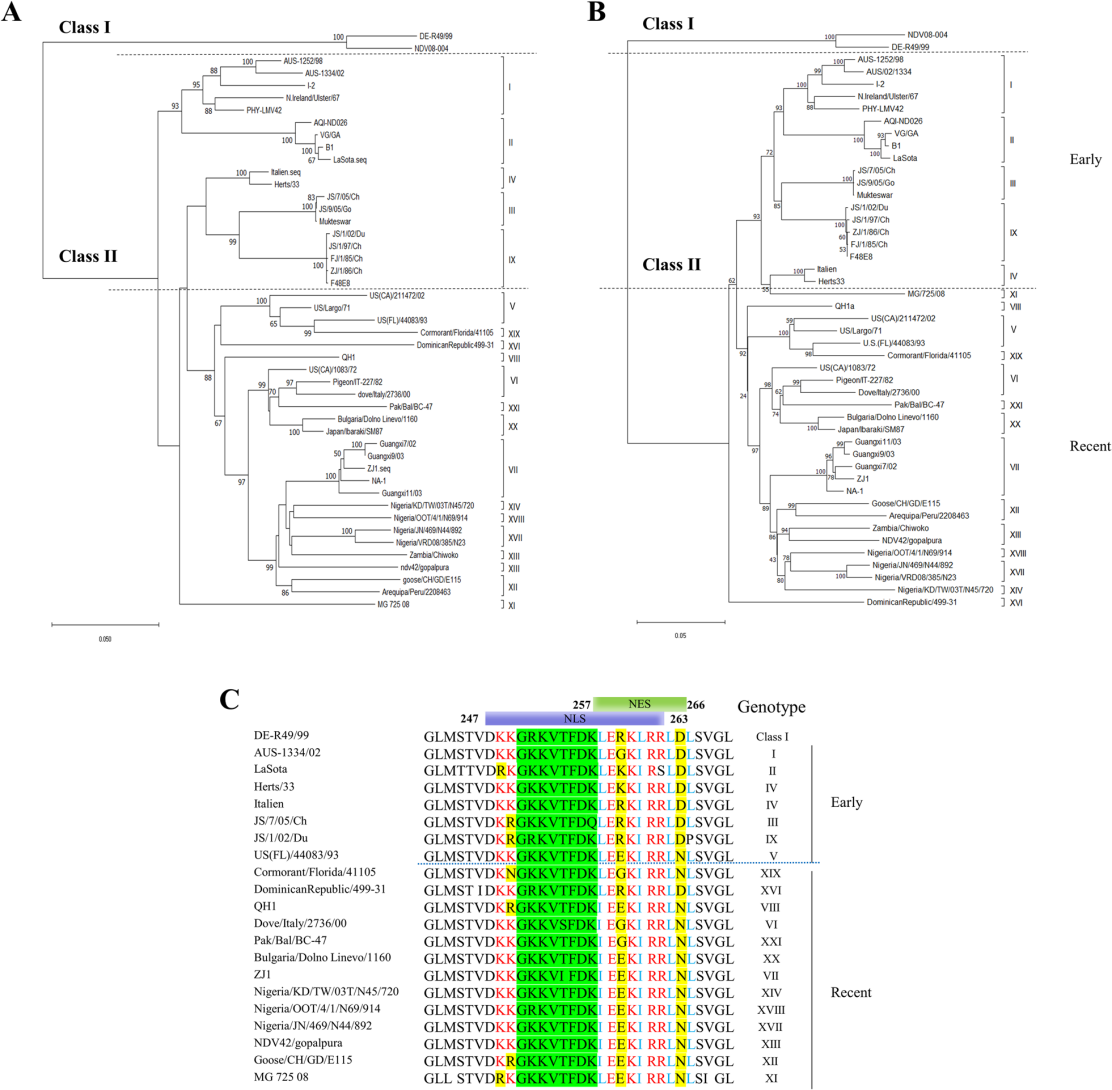
**The amino acid sequences of M protein’s NES**^**257–266**^** motif are linked to the evolution history of NDV.** Phylogenetic tree constructed using the complete nucleotide sequences of the fusion gene of representative isolates of the M gene (**A**) or F gene (**B**) of viruses representing NDV belonging to different genotypes respectively. The tree was constructed using the Neighbor-Joining method and the numbers at the nodes are evaluated by bootstrap analysis (1000 replicates). Only values above 50% are shown. The tree is drawn to scale, with branch lengths measured in the number of substitutions per site. The analysis involved 47 nucleotide sequences. Evolutionary analyses were conducted in MEGA11. **C** To compare the amino acid sequences of NES motifs in M proteins of different NDV genotypes. Areas covering NLS and NES sequences are labeled. Conserved sequences in NLS are labeled with green background, iconic leucine and isoleucine residues in NES are labeled with blue, amino acid residues at conserved sites are labeled with red, and amino acid residues at mutation-prone sites are labeled with yellow background.

In our previous report, we confirmed the existence of a classical NES sequence on M protein (i.e., aa 257 to 266), which plays a very important role in the nuclear export of M protein [23]. It is worth noting that there are differences in the NES sequences of M proteins amongst various NDV genotypes. To confirm this observation, the representative NDV strains of different genotypes were collected from GenBank and the phylogenetic tree was constructed based on the nucleotide sequences of their M protein (Figure 1A and Additional file 1). It is a well-established fact that the six viral genes within the NDV's single-negative-stranded RNA genome are subject to co-evolution [4, 6]. The presented phylogenetic tree conclusively demonstrates that the clustering patterns of NDV strains in the current analysis closely align with those observed in the tree derived from the F gene sequences (Figure 1B). In these trees, NDV isolates were classified into two major clades, Class I and Class II [4, 44, 45]. Class I and the genotype I within Class II are predominantly composed of non-virulent isolates from wild waterfowl and poultry species globally. These isolates may have been prevalent in birds for a long time. The NDV strains belonging to the I-IV and IX genotypes within Class II are early lineage that date back to before 1960 genotype. In contrast, the V-VIII genotypes are considered recent lineage, having emerged after 1960 and characterized by a genome size of 15,192 nt [45–49]. Among these genotypes, the V and VI genotypes were isolated during the second pandemic, which occurred in the 1960s-70 s. The genotypes VIII and VII, which triggered the fourth and most recent pandemic, emerged in the late 1980 s across the Far East, Europe, and South Africa [15, 45, 50, 51]. With the evolution of the viruses and the advancement of genotyping methodologies, more genotypes, including XI-XXI, have been identified in recent years (genotype XV that contains only recombinant sequences was excluded from the final analyses). The genotypes XXI, XIX, and XX, show higher homology with genotypes V and VI [3, 4]; the other genotypes, including XI-XIV, XVII, and XVIII display higher homology with genotype VII [3, 4, 7]. These genotypes are classified into the recent lineage in this study (Figure 1).

We conducted a comparative analysis of the NES motif in the M protein across all genotypes of NDV, and found that, despite the motif's conservation among genotypes, there are notable variations in the amino acid sequences of NDV strains that belong to early and recent lineages. Early NDV strains generally displayed an NES motif with the sequence ^257^LEGK/RIRRLDL^266^, featuring predominantly alkaline amino acids lysine (K) or arginine (R) at the 259^th^ position and an acidic aspartic acid (D) at the 265^th^ position. In contrast, the amino acids at positions 259 and 265 within the NES motif of NDVs from the recent lineage were altered, with glutamic acid (E) commonly observed at position 259 and asparagine (N) at position 265 in most genotypes (Figure 1C). Furthermore, certain genotypes and strains exhibited specific mutations within their NES motifs. For instance, the M protein of the LaSota strain from genotype II contained a unique mutation at the 263^rd^ amino acid position, where serine (S) substituted for arginine (R). This substitution is known to impede the nuclear export efficiency of the M protein and thereby retard the propagation of NDV [23]. At position 259 of the NDV M protein, glycine (G) was found in some strains from genotypes I, VI, XVIII, and XIX, possibly indicating a genetic connection between these genotypes. Additionally, the M protein of the early and recent lineages display a difference at amino acid 257. Early strains typically have leucine (L) at this site, whereas the majority of recent strains carry isoleucine (I) at position 257, with only a few retaining leucine (L). These findings suggest that the amino acid sequences within the M protein's NES motif are intricately linked to the evolutionary history of NDV.

### The NES^257−266^-mediated nuclear export efficiency of M proteins varies among different genotypes

**Figure 2:**
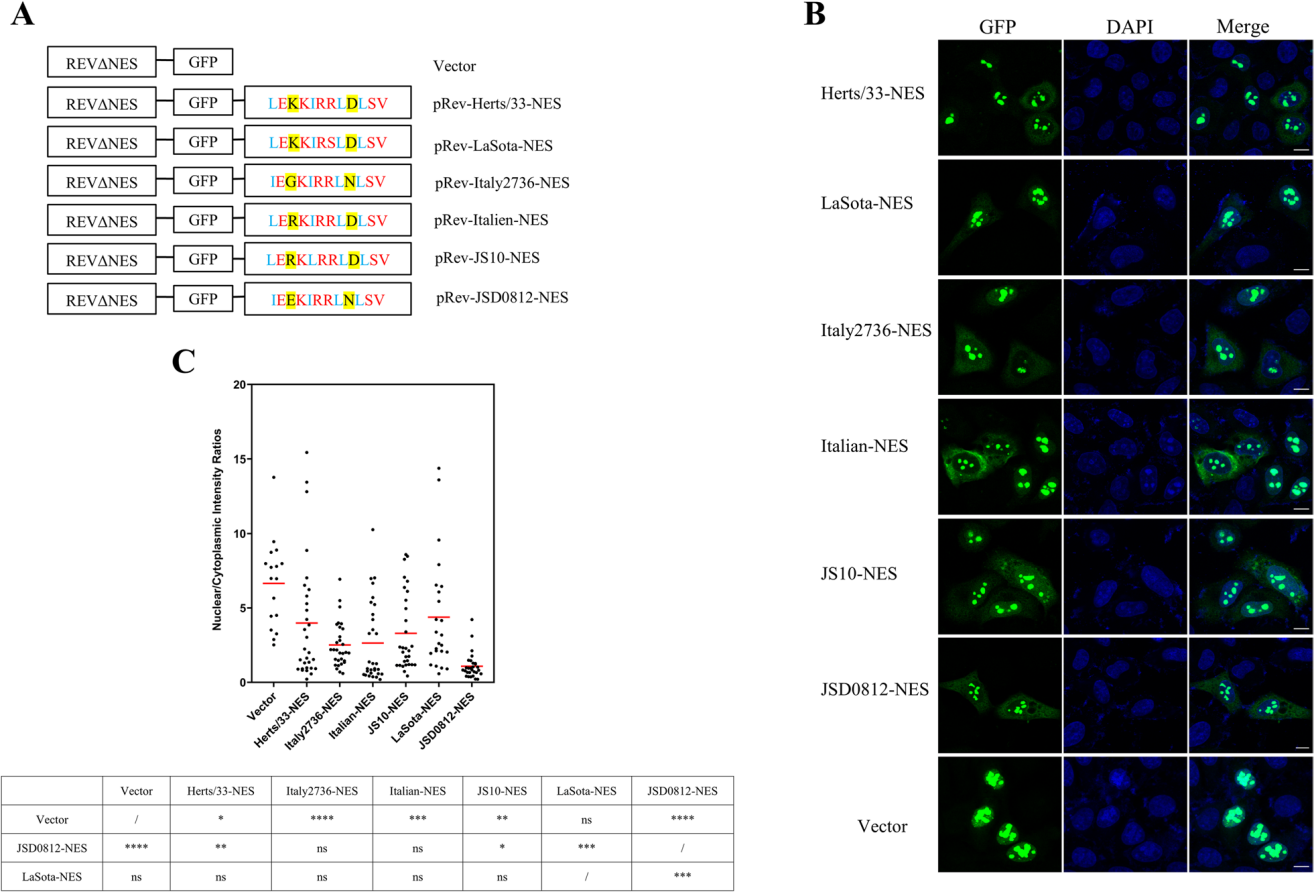
**The NES**^**257–266**^**-mediated nuclear export efficiency of M proteins varies among different genotypes.** **A** The reporter plasmid Rev(1.4)-GFP was utilized for NES detection and the schematic diagram for the mutants were aligned. Rev(1.4)-GFP encodes a GFP-fused HIV Rev protein mutant in which the only NES motif is deleted but its NLS motif is preserved. Herts/33, LaSota, Italy2736, Italien, JS10 and JSD0812 M protein NES were synthesized and fused to the C-terminal end of GFP, and the iconic leucine and isoleucine residues in NES were labeled with blue characters, the conserved amino acid residue sites were labeled with red characters, and the mutation-prone amino acid residue sites were labeled with yellow background. **B** Recombinant pRev(1.4)-GFP plasmid with different NES was transfected into Hela cells instantaneously. **C** The N:C ratio for each mutation was determined and shown in a scatter diagram. The arithmetic mean of N:C ratios for each sample is marked by a red line. The N: C ratio was compared statistically with that of pRev(1.4)-GFP (vector) by ANOVA with Bonferroni correction for multiple comparisons. The *P* values were both shown on the top of the scatter diagram (*P < 0.05; **P < 0.01; ***P < 0.001; ****P < 0.0001; ns, not significant).

Upon analyzing the NES^257−266^ motif sequences across all NDV genotypes, we identified a total of six distinct NES motifs that are naturally present. In the context of this study, these motifs have been named after their respective representative strains, referred to as Herts/33-NES, Italy2736-NES, Italian-NES, JS10-NES, LaSota-NES, and JSD0812-NES (Figure 2A). To assess the functional differences among these NES motifs, the recombinant plasmids containing these NES motifs in the C-terminal of Green fluorescent protein (GFP) were constructed respectively using the reporter plasmid Rev(1.4)-GFP as previously described [23] (Figure 2A). The subcellular localization of these mutants was examined under immunofluorescence microscopy, and quantification of the nucleus-to-cytoplasm ratio (N:C) was performed. As shown in Figure 2B, the recombinant GFP (rGFP) expressed by the negative control, pRev(1.4)-GFP is confined exclusively to the cell nucleus. In contrast, the NDV NES motifs promote varying levels of nuclear export for the rGFP. The N:C ratio presented in Figure 2C clearly demonstrates significant disparities in the overall nuclear export of the GFP protein mediated by these NES motifs. Among the motifs examined, the JSD0812-NES from the NDV strains of the recent lineage exhibited the highest nuclear export capability, with the rGFP predominantly localized in the cytoplasm. Statistical analysis revealed that the N:C ratio facilitated by JSD0812-NES was exceptionally low, at 1.0 ± 0.9, significantly less than that observed for the negative control, pRev(1.4)-GFP (*P* < 0.0001) (Figure 2C). Conversely, the LaSota-NES and Herts/33-NES from the NDV strains of the early lineage were less effective, leading to a notable concentration of rGFP within the nucleus, similar to the negative control, with N:C ratios of 4.4 ± 3.8 and 4 ± 4, respectively (Figure 2B). The other NES motifs, including Italy2736-NES and Italian-NES, exhibited N:C values of 2.5 ± 1.5 and 2.6 ± 2.6, respectively, which were significantly higher than those of JSD0812-NES but still significantly lower than the early lineage NESs. These NES motifs originate from NDV genotypes that are in the transitional phase between the early and recent lineages (Figure 1), and they exhibit nuclear export capabilities that are intermediate between those of the recent and early lineage NES motifs. Furthermore, the sequences of JS10-NES and Italien-NES differ by a single amino acid, a substitution from isoleucine (I) to leucine (L) at position 261, which is exclusive to Class I strains. It is shown that this I261L mutation has negligible effect on the NES's capability in nuclear export, and there is no statistically significant difference in the nuclear export efficacy between JS10-NES and the Italy2736-NES or Italian-NES. These findings suggest that the nuclear export mediated by the M proteins varies depending on the NES motif types, with the JSD0812-NES of the recent lineage showing the most effective nuclear export capability.

### Site-specific amino acids in NES motifs and their Impact on nuclear export efficacy

**Figure 3:**
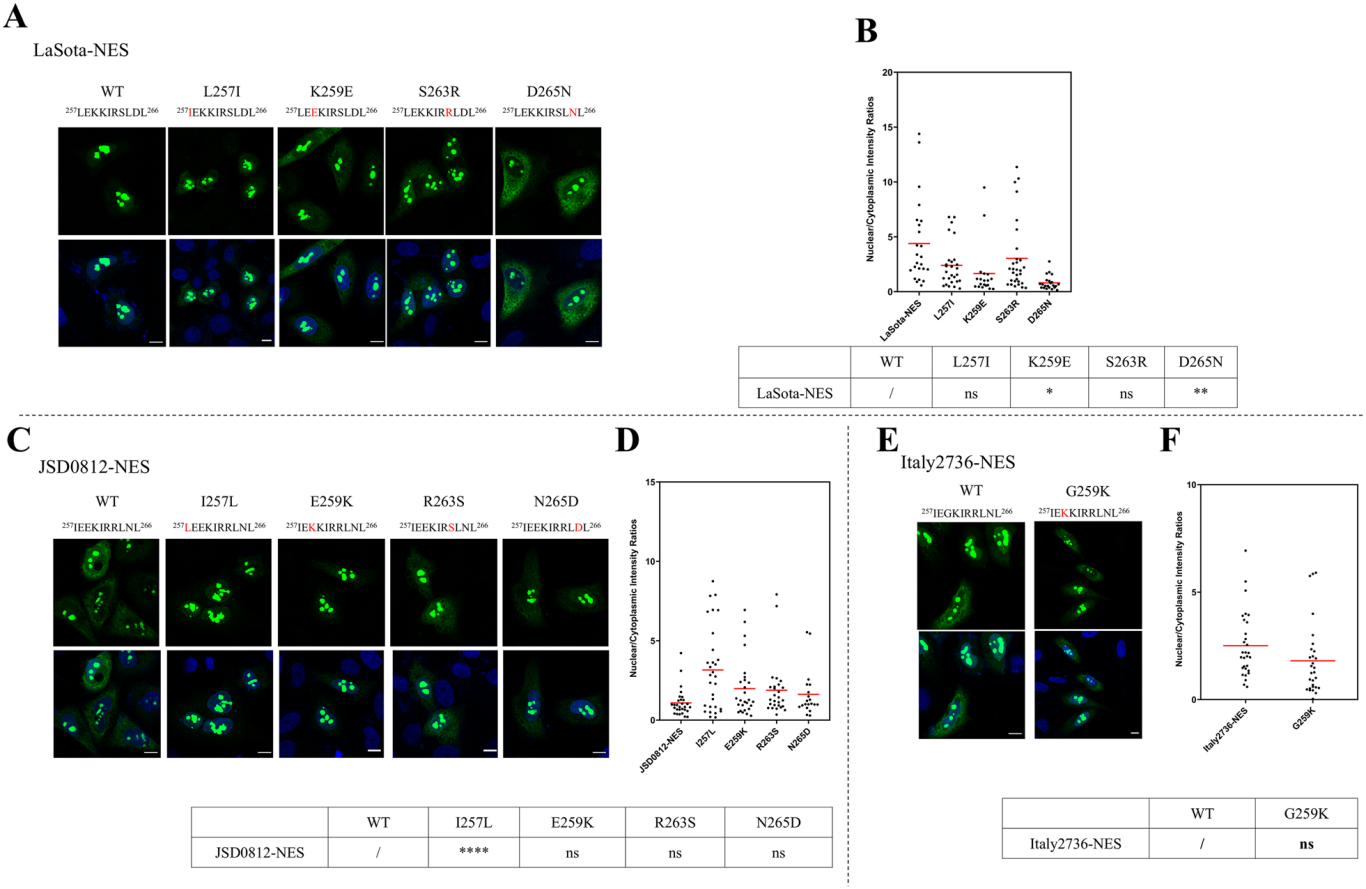
**Site-specific amino acids in NES**^**257–266**^** motifs have different effects on its ability to mediate protein nuclear export.** **A** Leucine (L), lysine (K), serine (S) and aspartic acid (D) of pRev(1.4)-LaSota-NES were replaced by isoleucine (I), glutamic acid (E), arginine (R) and aspartic acid (N) at residues 257, 259, 263 and 265, respectively. The recombinant plasmids pRev(1.4)-GFP-LaSota-NES-L247I, pRev(1.4)-GFP-LaSota-NES-K259E, pRev(1.4)-GFP-LaSota-NES-S263R and pRev(1.4)-GFP-LaSota-NES-D265N were constructed. All mutants were transfected into HeLa cells for NES experiments. 24 h after transfection, the cells were fixed and stained with DAPI to reveal the nucleus. Green and blue fluorescence were observed under a fluorescence microscope. **B** The N:C ratio for each mutation was determined and shown in a scatter diagram. The arithmetic mean of N:C ratios for each sample is marked by a red line. The N: C ratio was compared statistically with that of pRev(1.4)-GFP-LaSota-NES by ANOVA with Bonferroni correction for multiple comparisons. The *P* values were both shown on the top of the scatter diagram (*, *P* < 0.05; **, *P* < 0.01; ***, *P* < 0.001; ****, *P* < 0.0001; ns, not significant). **C** Isoleucine (I), glutamic acid (E), arginine (R) and aspartic acid (N) of pRev(1.4)-JSD0812-NES were replaced by Leucine (L), lysine (K), serine (S) and aspartic acid (D) at residues 257, 259, 263 and 265, respectively. The recombinant plasmids pRev(1.4)-GFP-JSD0812-NES-I247L, pRev(1.4)-GFP-LaSota-NES-E259K, pRev(1.4)-GFP-LaSota-NES-R263S and pRev(1.4)-GFP-LaSota-NES-N265D were constructed. All mutants were transfected into HeLa cells for NES experiments. 24 h after transfection, the cells were fixed and stained with DAPI to reveal the nucleus. Green and blue fluorescence were observed under a fluorescence microscope. **D** The N:C ratio for each mutation was determined and shown in a scatter diagram. The arithmetic mean of N:C ratios for each sample is marked by a red line. The N: C ratio was compared statistically with that of pRev(1.4)-GFP-JSD0812-NES by ANOVA with Bonferroni correction for multiple comparisons. The *P* values were both shown on the top of the scatter diagram (*P < 0.05; **P < 0.01; ***P < 0.001; ****P < 0.0001; ns, not significant). **E** Glycine (G) of pRev(1.4)-GFP-Italy2736-NES were replaced by lysine (K), at residues 259. The recombinant plasmids pRev(1.4)-GFP-Italy2736-NES-G247K were constructed. All mutants were transfected into HeLa cells for NES experiments. 24 h after transfection, the cells were fixed and stained with DAPI to reveal the nucleus. Green and blue fluorescence were observed under a fluorescence microscope. **F** The N:C ratio for each mutation was determined and shown in a scatter diagram. The arithmetic mean of N:C ratios for each sample is marked by a red line. The N: C ratio was compared statistically with that of pRev(1.4)-GFP-Italy2736-NES by ANOVA with Bonferroni correction for multiple comparisons. The *P* values were both shown on the top of the scatter diagram (*P < 0.05; **P < 0.01; ***P < 0.001; ****P < 0.0001; ns, not significant).

The NES^257−266^ motif of JSD0812-NES mediates the nuclear export of the M protein more effectively than LaSota-NES, with four amino acid mutations observed, including L257I, K259E, S263R, and D265N. To determine which of these amino acid sequences are crucial for enhancing the nuclear export of the M protein, we first introduced mutations at these four sites on the wild type (wt) reporter plasmid pRev(1.4)-GFP-LaSota-NES, creating constructs pRev(1.4)-LaSota-NES-L257I, K259E, S263R, and D265N, and assessed their impact on the nuclear export efficiency of rGFP. As shown in Figures 3A and B, mutations at all four sites increased the nuclear export efficiency of rGFP, with the mutations K259E (N:C ratio of 0.8 ± 0.6) and D265N (N:C ratio of 0.8 ± 0.7) having significant effects (*P* < 0.01) (Figure 3B). Subsequently, we performed opposite mutations on the wt pRev(1.4)-JSD0812-NES plasmid and obtained constructs pRev(1.4)-JSD0812-NES-I257L, E259K, R263S, and N265D. Figure 3C demonstrates that mutations at these four sites all reduced the nuclear export efficiency of rGFP, with the mutation I257L (N:C ratio of 3.2 ± 2.6) being particularly significant (*P* < 0.0001) (Figures 3C and D). Furthermore, considering that some NDV strains’ M protein has a K to G mutation at position 259, we made a G259K mutation in Italy2736-NES, and the results showed that this site did not have a significant impact on the nuclear export efficiency of the M protein (Figures 3E and F). In summary, the differences in amino acids at positions 257, 259, and 265 in the M protein of early and recent NDV strains have the most pronounced effect on the NES motif's nuclear export efficiency.

### Enhanced nuclear export of LaSota M protein through K259E, S263R, and D265N mutations

**Figure 4:**
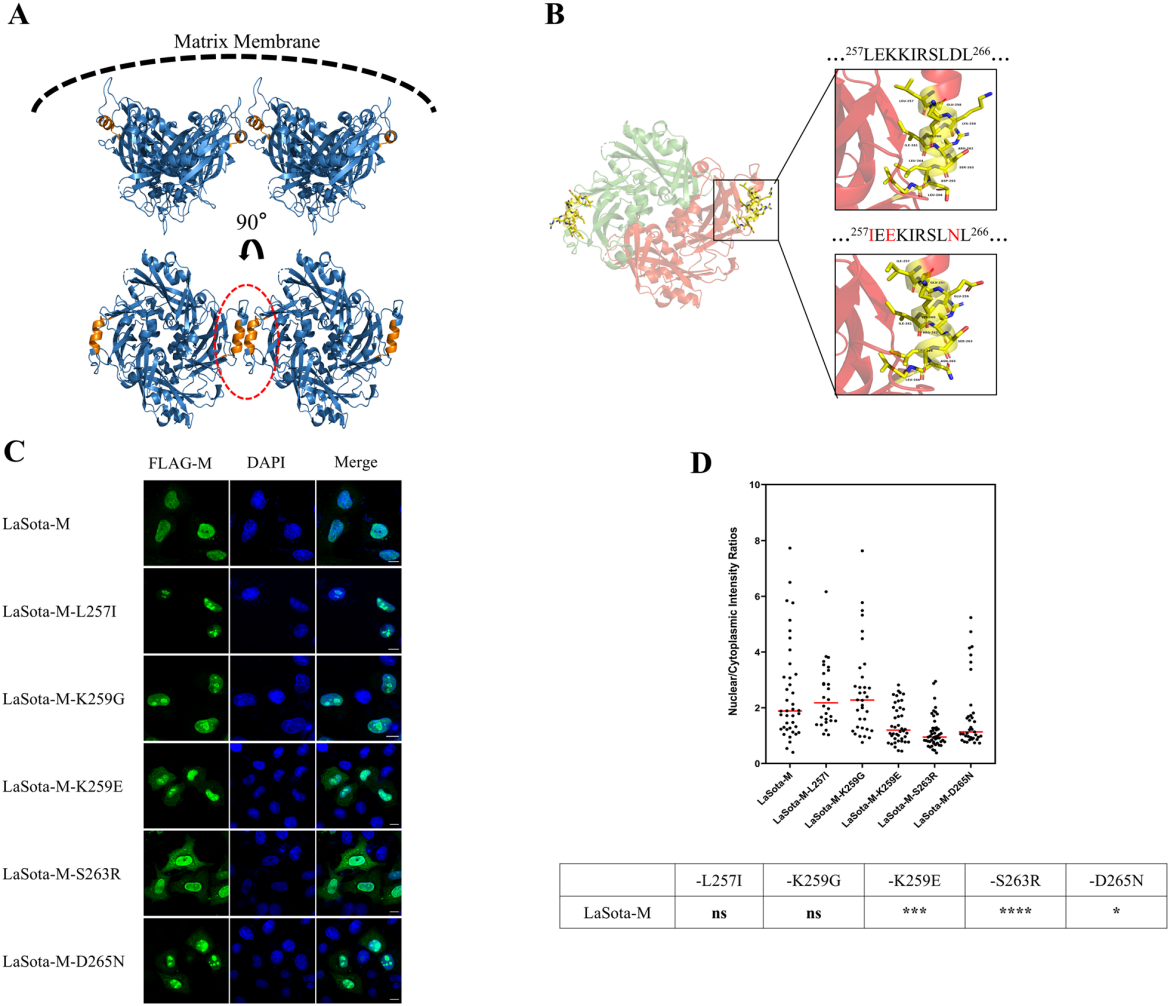
**Site-specific amino acids in NES**^**257–266**^** motifs have different effects on the nuclear ability of LaSota M protein.** **A** Structural model of NDV-M protein dimer generated using PyMOL (version 3.1) based on the crystal structure solved previously (PDB: 4G1G). Two M protein protomers in dimer are shown in blue and the NES motifs are shown in orange. NES motifs were in the interface between every two M protein dimers. The dashed black line demarcates the membrane and matrix layer interface and illustrates the membrane curvature generated by the matrix protein array, according to a previous report. **B** The spatial structure changes of M protein at different sites in the mutant NES motif were shown. **C** Leucine (L), lysine (K), serine (S) and aspartic acid (D) of pCMV-LaSota-M-3×flag were replaced by isoleucine (I), glutamic acid (E)/Glycine (G), arginine (R) and aspartic acid (N) at residues 257, 259, 263 and 265, respectively. The recombinant plasmids pCMV-LaSota-M-L257I-3×flag, pCMV-LaSota-M-K259E-3×flag, pCMV-LaSota-M-K259G-3×flag, pCMV-LaSota-M-S263R-3×flag and pCMV-LaSota-M-D265N-3×flag were constructed. All mutants were transfected into HeLa cells for NES experiments.24 h after transfection, the cells were observed by indirect immunofluorescence. **D** The N:C ratio for each mutation was determined and shown in a scatter diagram. The arithmetic mean of N:C ratios for each sample is marked by a red line. The N: C ratio was compared statistically with that of pCMV-LaSota-M-3×flag by ANOVA with Bonferroni correction for multiple comparisons. The *P* values were both shown on the top of the scatter diagram (*P < 0.05; **P < 0.01; ***P < 0.001; ****P < 0.0001; ns, not significant).

Based on the crystal structure of the NDV M protein, it is established that the M protein exists as a dimer, which serves as the fundamental building block for the subsequent assembly into a tetrameric structure. The inter-dimer contacts are pivotal in determining the curvature of the assembled matrix array, thereby shaping the virion’s morphology during budding [19, 52]. As depicted in Figure 4A, the NES motif is located at the dimer interface, adjacent to the region that interacts with the negatively charged membrane surface. This unique structural locale underscores its critical role in the protein's trafficking and the process of virus budding. The amino acid substitution observed between the LaSota-NES and JSD0812-NES sequences have led to structural changes in this region (Figure 4B). Notably, mutations involving acidic-basic amino acids, such as K259E and D265N, will lead to direct changes in charge distribution within this domain (data not shown). We introduced mutations to the amino acid residues of interest within the eukaryotic expression plasmid wt L-M, which stably expresses the LaSota M protein, yielding the recombinant plasmids L-M-L257I, -K259G, -K259E, -S263R, and -D265N. Subsequent analysis of the distribution of these recombinant M proteins between the nucleus and cytoplasm confirmed that the L257I and K259G mutations do not alter the nuclear export of the LaSota M protein. In contrast, the mutations at the K259E, S263R, and D265N positions all enhance the nuclear export of the LaSota M protein (Figures 4C and D).

### Comparative analysis of nuclear export of M proteins mediated by NES motif and K247 residue

**Figure 5:**
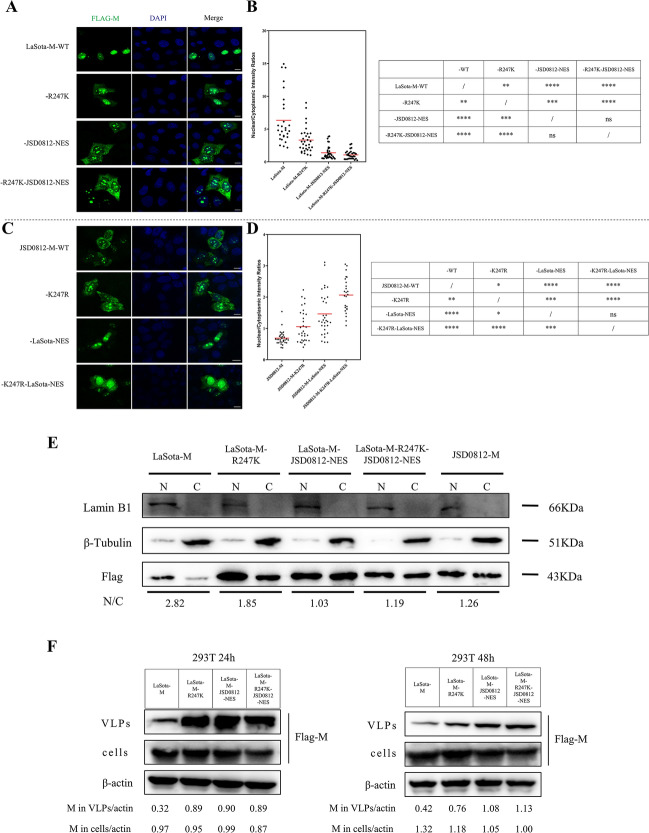
**Comparative analysis of nuclear export of M proteins mediated by NES**^**257–266**^** Motif and K247R mutation.** **A** Arginine (R) and LaSota-NES motif of pCMV-LaSota-M-3×flag were replaced by lysine (K) and JSD0812-NES at residues 247 and 257–266, respectively. The recombinant plasmids pCMV-LaSota-M-R247K-3×flag, pCMV-LaSota-M-JSD0812-NES-3×flag and pCMV-LaSota-M-R247K-JSD0812-NES-3×flag were constructed. All mutants were transfected into HeLa cells for NES experiments. 24 h after transfection, the cells were observed by indirect immunofluorescence. Green and blue fluorescence were observed under a fluorescence microscope. **B** The N:C ratio for each mutation was determined and shown in a scatter diagram. The arithmetic mean of N:C ratios for each sample is marked by a red line. The N:C ratio was compared statistically with that of pCMV-LaSota-M-3×flag by ANOVA with Bonferroni correction for multiple comparisons. The *P* values were both shown on the top of the scatter diagram (*P < 0.05; **P < 0.01; ***P < 0.001; ****P < 0.0001; ns, not significant). **C** Lysine (K) and JSD0812-NES motif of pCMV-JSD0812-M-3×flag were replaced by Arginine (R) and LaSota-NES at residues 247 and 257–266, respectively. The recombinant plasmids pCMV-JSD0812-M-K247R-3×flag, pCMV-JSD0812-M-LaSota-NES-3×flag and pCMV-JSD0812-M-K247R-LaSota-NES-3×flag were constructed. All mutants were transfected into HeLa cells for NES experiments. 24 h after transfection, the cells were observed by indirect immunofluorescence. Green and blue fluorescence were observed under a fluorescence microscope. **D** The N:C ratio for each mutation was determined and shown in a scatter diagram. The arithmetic mean of N:C ratios for each sample is marked by a red line. The N: C ratio was compared statistically with that of pCMV-JSD0812-M-3×flag by ANOVA with Bonferroni correction for multiple comparisons. The *P* values were both shown on the top of the scatter diagram (*P < 0.05; **P < 0.01; ***P < 0.001; ****P < 0.0001; ns, not significant). **E** Cytoplasmic and nuclear M proteins in this experiment were separated and compared in WB assay. The amounts of M protein in the nuclei and cytoplasm were quantified using ImageJ software, and then the N:C ratios of M proteins were calculated and labeled under the bands. **F** 293 T cells were transfected with plasmids expressing FLAG-tagged LaSota-M protein and derived mutants. The amounts of VLPs in the supernatants and cells at 24 and 48 hpt were detected in the WB assay and then quantified using ImageJ software.

Given that each of the four point mutations, including L257I, K259E, S263R, and D265N, in the JSD0812-NES^257−266^ motif can significantly enhance the nuclear export efficiency of LaSota-NES^257−266^, we introduced these mutations into the eukaryotic expression plasmid of the LaSota M protein (LaSota-M), resulting in a new construct designated as LaSota-M -JSD0812-NES. As shown in Figure 5A, the wild-type LaSota M protein, expressed by the parental plasmid LaSota-M, was predominantly localized in the nucleus; in contrast, the recombinant LaSota M protein, expressed by the LaSota-M-JSD0812-NES plasmid carrying the JSD0812-NES motif, was effectively exported to the cytoplasm. This function of the JSD0812-NES motif is strikingly similar to that of the ubiquitination site K247 of the M protein, as previously reported [23]. By comparison, the JSD0812-NES exhibits a significantly higher capacity for promoting nuclear export (N:C ratios of 1.4 ± 1) compared to the R247K mutation (Figures 5A and B). The efficacy of the JSD0812-NES motif was further confirmed through the use of the eukaryotic expression plasmid JSD0812-M (Figure 5C). This plasmid expresses the M protein from the genotype VII JSD0812 strain, which is characterized by its strong nuclear export capability. Upon mutating the NES motif to the LaSota-NES sequence, there was a significant suppression of the M protein’s nuclear export, with the inhibitory impact being notably more pronounced than that caused by the K247R mutation (Figure 5D). Moreover, the synergistic effect of the K247R and JSD0812-NES motifs on promoting nuclear export is evident in the JSD0812-M plasmid but less pronounced in the LaSota-M plasmid, suggesting that other factors may also influence the nuclear export of M proteins.

To further determine the effect of NES motif and K247 residue on nuclear localization, the M proteins were separated from the nuclear and cytoplasmic fractions of cells that had been transfected with the plasmids LaSota-M, LaSota-M-R247K, LaSota-M-JSD0812-M-NES, LaSota-M-R247K-JSD0812-M-NES, and JSD0812-M. Subsequent quantification via Western blot analysis (Figure 5E) revealed that both the substitution of the NES motif and the R247K mutation potently enhanced the relative abundance of M protein in the cytoplasm, while also significantly elevating the total protein levels. Notably, there was no evidence of a synergistic effect when the NES motif replacement and the R247K mutation were combined in the LaSota-M plasmids. These findings are in agreement with the outcomes from our previous IFA assay, indicating that the JSD0812-NES exhibits a markedly greater capacity to induce nuclear export compared to the LaSota-NES. Moreover, this enhanced nuclear export capacity appears to be independent of the activity of the ubiquitination site at position 247.

Since NDV-M is known to form VLPs by itself and bud off the cell surface spontaneously, it is reasonable to deduce that improving the nuclear export efficiency of the M protein mediated by the NES^257−266^ motif could enhance the M protein’s capability to yield VLPs. We detected the VLP amounts of the LaSota-M protein with that of the mutants LaSota-M-R247K, LaSota-M-JSD0812-NES, and LaSota-M-R247K-JSD0812-NES after transfection. In brief, the supernatant was collected at 24 and 48 hpt, concentrated to 100 μL, and then analyzed using WB detection. The results revealed that the VLP production by the three M protein mutants was significantly higher than that of the parental LaSota-M protein at both 24 and 48 hpt (Figure 5F), the recombinant M protein with the JSD0812-NES mutation exhibited a higher level of VLP production among the three mutants. Besides, these findings indicate that the NES and R247K mutations do not exert a synergistic effect on the nuclear export of the LaSota M protein, a conclusion that aligns with the results of previous IFA (Figures 5A–D) and nuclear-cytoplasmic separation experiments (Figure 5E).

### Nuclear export of the M protein depends on the CRM1 nuclear export pathway

**Figure 6:**
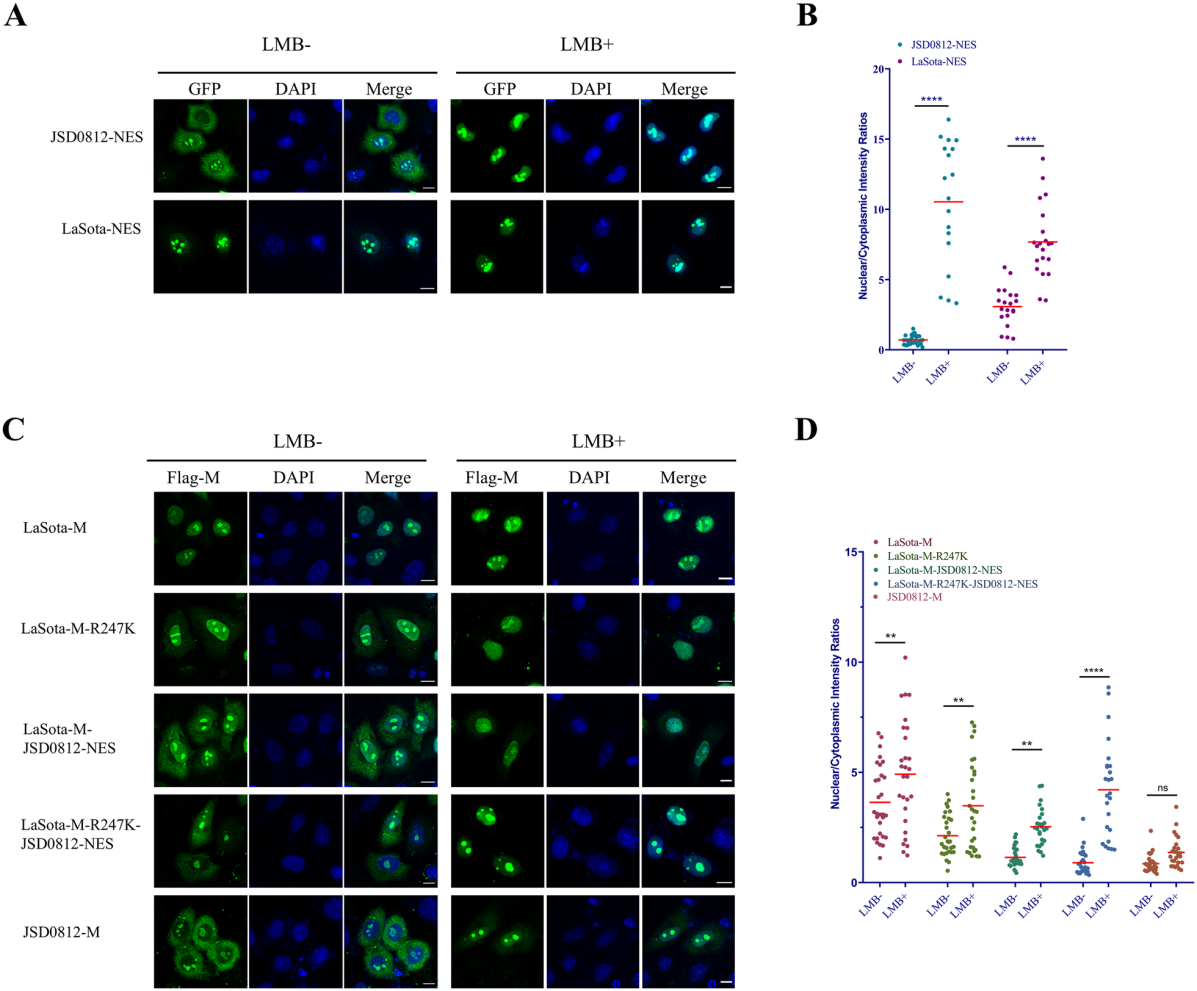
**The NES**^**257–266**^** motif facilitates the nuclear export of the M protein through the CRM1-dependent pathway.** **A** The reporter plasmids Rev(1.4)-GFP-LaSota-NES, Rev(1.4)-GFP-JSD0812-NES were transfected into Hela cells and treated with or without LMB (10 ng/mL) for 3 h. The cells were observed by indirect immunofluorescence. **B** The N:C ratio for each mutation was determined and shown in a scatter diagram. The arithmetic mean of N:C ratios for each sample is marked by a red line. The N: C ratio was compared statistically with the untreated group by ANOVA with Bonferroni correction for multiple comparisons. The *P* values were both shown on the top of the scatter diagram (*P < 0.05; **P < 0.01; ***P < 0.001; ****P < 0.0001; ns, not significant). **C** pCMV-LaSota-M-3×flag, pCMV-JSD0812-M-3×flag, PCMV-LaSota-M-R247K-3×flag, PCMV-LaSota-M-JSD0812-NES-3×flag, pCMV-LaSota-M-3×flag, PCMV-LaSota-M-3×flag and R247K-JSD0812-NES-3×flag were transfected into HeLa cells for NES assay. After transfection for 18 h, cells were treated with or without LMB for 3 h, and then the cells were observed by indirect immunofluorescence. **D** The N:C ratio for each mutation was determined and shown in a scatter diagram. The arithmetic mean of N:C ratios for each sample is marked by a red line. The N: C ratio was compared statistically with the untreated group by ANOVA with Bonferroni correction for multiple comparisons. The *P* values were both shown on the top of the scatter diagram (*P < 0.05; **P < 0.01; ***P < 0.001; ****P < 0.0001; ns, not significant).

Extensive research has established that the host cell’s nuclear export receptor, CRM1, selectively recognizes and interacts with the classical leucine/isoleucine-enriched nuclear export signals (NES) of the cargo proteins, thereby facilitating their nuclear export. In this study, an inhibitor of CRM1, leptomycin B (LMB) [53, 54], was used to block its nuclear exit pathway and to determine whether the NDV M protein depends on the CRM1-mediated nuclear export pathway for its own nuclear export. Firstly, we assessed the impact of LMB on the reporter plasmids pRev (1.4)-GFP-LaSota-NES and pRev (1.4)-GFP-JSD0812-NES. Upon fluorescence microscopy examination, it was observed that the JSD0812-NES in the untreated control group mediated robust export of rGFP from the nucleus. In stark contrast, treatment with LMB significantly confined the rGFP within the nucleus (Figure 6A). Furthermore, while the nuclear export of M protein by LaSota-NES was not apparent in the untreated group, fluorescence intensity analysis revealed the presence of some rGFP protein in the cytoplasm, with a N:C ratio of 3.0 (Figure 6B). Post-treatment with LMB, the ratio of rGFP in the nucleus to the cytoplasm was notably improved, increasing to a N:C ratio of 7.6, suggesting that the inhibition of CRM1 by LMB effectively impedes the nuclear export promoted by NES motifs derived from NDV M proteins.

We then conducted a study to examine the effect of LMB on the nuclear export of the M protein, utilizing five eukaryotic expression plasmids: LaSota-M-Flag, LaSota-M-R247K-FLAG, LaSota-M-J-NES-FLAG, LaSota-M-R247K-J-NES-FLAG, and JSD0812-M-FLAG. The results indicated that, as expected from previous studies with reporter plasmids, LMB treatment effectively inhibited the export of the M protein from the nucleus (Figures 6C and D). Since the LaSota-NES is weak, the N:C ratio for the wild-type LaSota-M-Flag was relatively high, and this ratio was significantly increased after LMB treatment. Both the R247K and JSD0812-NES mutations were found to enhance the export efficiency of the LaSota M protein from the nucleus and significantly reduce the N:C ratio. In contrast, both the R247K and JSD0812-NES mutations failed to mediate effective nuclear export in the presence of LMB. Furthermore, LMB treatment also inhibited the nuclear export of the wild-type JSD0812-M-FLAG (Figures 6C and D). These data suggest that the nuclear export of M proteins, mediated by both the R247K and NES motif, is CRM1-dependent.

### The NES motif’s influence on the replication and virulence of NDV

**Figure 7:**
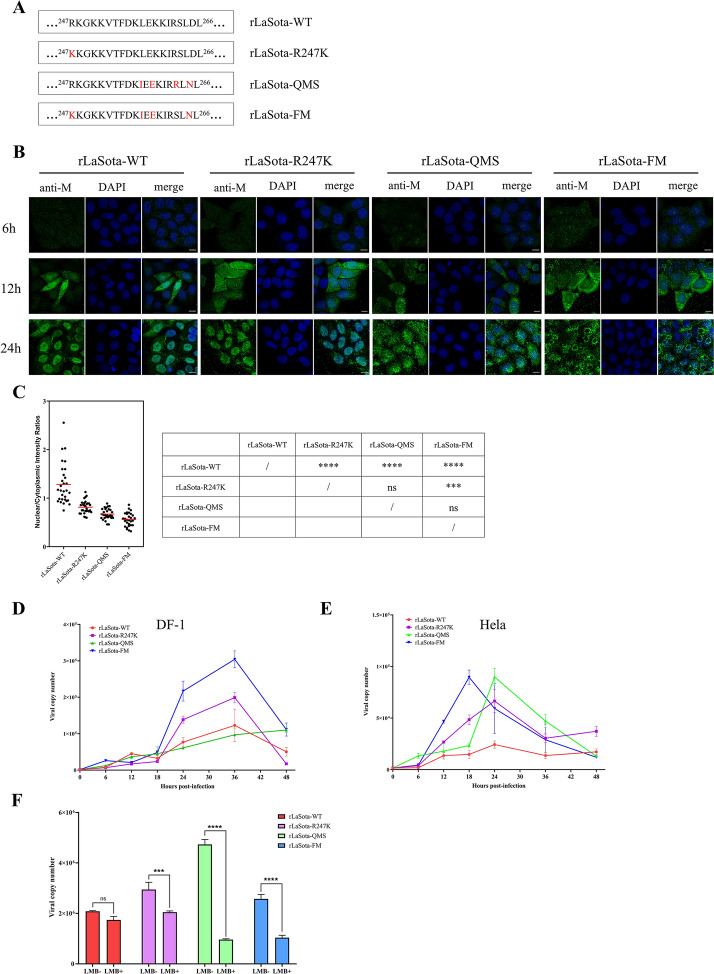
**The NES motif’s influence on the replication and virulence of NDV.** **A** Schematic representation of mutations of recombinant NDV. The arginine at the M protein 247 site of LaSota wild strain was mutated to lysine, and the rescued recombinant virus was named rLaSota-R247K. Leucine, lysine, serine and aspartic acid were mutated to isoleucine, glutamic acid, arginine and aspartic acid at sites 257, 259, 263, 265 of LaSota wild strain M protein, and the rescued recombinant virus was named rLaSota-QMS. On the basis of four amino acid sites mutation of rLaSota-QMS strain, arginine at site 247 was mutated to lysine, and a total of five mutation sites were introduced compared with the wild LaSota strain. The rescued recombinant virus was named rLaSota-FM. **B** Nuclear-cytoplasmic trafficking of NDV-M proteins during live viral infection of recombinant NDV was further determined by anti-M polyantibodies serum. **C** The N:C ratio of fluorescence distribution in 12 h sample cells were statistically analyzed, the N:C ratio for each mutation was determined and shown in a scatter diagram. The arithmetic mean of N:C ratios for each sample is marked by a red line. The N: C ratio was compared statistically with that of rLaSota-wt by ANOVA with Bonferroni correction for multiple comparisons. The *P* values were both shown on the top of the scatter diagram (*P < 0.05; **P < 0.01; ***P < 0.001; ****P < 0.0001; ns, not significant). Growth curves of rLaSota-wt, -R247K, -QMS, and -FM were detected in DF1 (**D**) and HeLa (**E**) cells and determined with qPCR values. The supernatant was harvested at 6, 12, 18, 24, 36, and 48 hpi. The amount of virus in the supernatant was quantified with real-time PCR of the NP gene. **F** The effect of LMB on recombinant NDV strains were detected. Hela cells were infected with each recombinant virus at a dose of 5 MOI for 20 h and then treated with LMB. At 4 h after treatment, the samples were harvested for the real-time PCR assay. All values were compared statistically by ANOVA (*P < 0.05; **P < 0.01; ***P < 0.001; ****P < 0.0001; ns, not significant).

Since the M protein is the key component for viral assembly and budding, we further investigated whether NES mutations also impact NDV replication. In our previous study, a recombinant LaSota (rLaSota) strain, termed R247K, was constructed carrying the R247K point mutations in its M protein. In this study, we successfully generated two recombinant LaSota strains, termed QMS and FM. The QMS strain was constructed with a four-point mutation, including L257I, K259E, S263R, and D265N in its M protein, while the FM strain was generated with a five-point mutation, including R247K, L257I, K259E, S263R, and D265N (Figure 7A). This means that the NES of the M protein in the rLaSota-QMS strain was mutated to the JSD0812-NES motif, and the strain rLaSota-FM possesses both the JSD0812-NES motif and the R247K mutation. In addition, the parental LaSota strain was also rescued, termed as rLaSoa-wt, serving as a control in this study. The cells were infected with these recombinant NDV strains, and the localization of the M protein within the cells was monitored at different time points. As depicted in Figure 7B, at 12 hpi, the M protein of the rLaSota-wt strain was predominantly found in the nucleus, whereas the M protein of the R247K mutant was distributed evenly between the nucleus and the cytoplasm. As expected, the M protein of the rLaSota-QMS, rLaSota-FM and rLaSota-R247K mutants was predominantly localized in the cytoplasm. Statistical analysis further revealed that the N:C ratio of the mutant M protein was significantly lower than that of the wild-type strain (Figure 7C). By 24 hpi, the rLaSota-QMS and rLaSota-FM recombinant mutants were more extensively distributed throughout the cells, whereas the M protein of the rLaSota-R247K and LaSota-wt strains was mainly located in the nucleus. These findings indicate that the JSD0812-NES motif, similar to the R247K mutation, has a pronounced effect on mediating the nuclear export of the M protein.

Then, we sought to determine the effect of NES substitution on viral replication efficiency. As shown in Table 1, the hemagglutinin (HA) titers, 50% egg infectious dose (EID_50_) titers, and Tissue Culture Infectious Dose 50 (TICD_50_) titers of the recombinant viruses QMS, R247K, and FM were all notably higher than those of the wt LaSota strain. To assess the growth kinetics of these viruses in cell culture, DF1 and HeLa cells were infected with rLaSota-wt, -R247K, -QMS, and -FM at a MOI of 5, respectively. Supernatants were collected at various time points for analysis, and growth curves were plotted. At the early stage of infection, the growth curves of the different strains in DF1 did not show significant differences. However, by 24 and 36 hpi, the viral titers of rLaSota-FM and -R247K were significantly elevated in DF1 cells, being 0.5 to 2 times higher than those of the wt strain, while no significant difference was observed between rLaSota-wt and -QMS viruses (Figure 7D). By contrast, the replication of the rLaSota-QMS strain was notably heightened in HeLa cells, particularly at 36 hpi (Figure 7E). Given that the leucine-rich nuclear export signal (NES) motif is frequently recognized by CRM-1 in mammalian cells, the substitution in this NES region may contribute to the enhanced infectivity of NDV in human cells. Collectively, these findings suggest that the K247R mutation and NES substitution do not hinder the growth of the recombinant viruses but rather can significantly enhance viral yield. This improvement in replication efficiency also contributes to the virus's increased virulence. As shown in Table 1, the MDT of the rLaSota-QMS, -R247K, and -FM strains were lower than that of the wt LaSota strain, and their ICPI values were higher. These results indicate that while enhancing the nuclear export efficiency of the M protein does not convert a non-virulent strain into a virulent one, it can still elevate the virus's virulence by enhancing the strain’s replication ability.

**Table 1:** **Results for fifth generation of recombinant viruses**

Virus	Log_2_HA		lgTCID_50_^e^	lgEID_50_^*b*^	MDT^*c*^	ICPI^*d*^
	Mean ± SEM^*a*^	Max				
La Sota-WT	10.39 ± 1.24	12	−8.29	−8.68	142.00	0.025
rLa Sota-R247K	11.03 ± 0.97	13	−8.41	−9.17	114.00	0.075
rLa Sota-QMS	11.28 ± 0.84	13	−8.43	−9.38	118.00	0.075
rLa Sota-FM	11.69 ± 0.59	13	−8.59	−9.17	115.20	0.1

^a^SEM, standard error of the mean.

^b^lgEID_50_, logarithm base 10 of 50% egg culture infective dose.

^c^MDT, mean death time.

^d^ICPI, intracerebral pathogenicity index.

^e^lgTCID_50_, logarithm base 10 of 50% tissue culture infectious dose.

In previous studies, we identified that the nuclear export of M proteins, mediated by either K247 ubiquitination or the NES^257−266^ motif, is dependent on CRM1 pathway. To further elucidate the impact of the CRM1 pathway on viral infection, rNDV-infected cells were treated with the CRM1 inhibitor, LMB. Briefly, four rLaSota strains were individually inoculated into HeLa cells at a dose of 5 MOI. After a 20-h incubation, the cells were treated with LMB for 4 h, and the virus titer in the infected cells was detected to determine the effect of LMB on viral replication. The results revealed that the wt rLaSota strain, which lacked both the R247K mutation and the NES^257−266^ motif replacement, was insensitive to LMB, suggesting that the M protein from wt LaSota may undergo nuclear export via a CRM1-independent way (Figure 7F). In contrast, LMB treatment significantly suppressed the growth of the rLaSota-R247K, rLaSota-QMS, and rLaSota-FM strains, indicating that the enhanced viral replication caused by the R247K mutation and the NES^257−266^ motif replacement in the M protein of rLaSota is associated with the cellular CRM1 pathway. Furthermore, the inhibitory effect of LMB was more pronounced in the rLaSota-QMS strain compared to the rLaSota-R247K strain, suggesting a higher dependence on the CRM1 pathway for the NES^257−266^ function.

## Discussion

The ongoing epidemic and transmission of NDV worldwide have led to a continuous process of mutation, resulting in the gradual emergence of an expanding array of genotypes [4]. These mutated strains may exhibit enhanced biological properties. As the virus becomes more virulent, it poses significant threats to the global poultry industry, potentially causing substantial economic losses. The classical method for assessing the virulence of NDV involves examining the cleavage site motif of its F protein. The WOAH defines virulent strains as those with an ICPI of 0.7 or higher, or those possessing a fusion cleavage site characterized by multiple basic amino acids and phenylalanine at position 117 [4]. In contrast, low-virulence NDV strains display monobasic fusion cleavage site motifs at amino acid positions 112–113 and 115–116, along with a leucine (L) at position 117 of the F protein [55]. These strains are only cleavable by trypsin-like enzymes present in the respiratory and intestinal tracts, thereby confining their replication to these systems [5]. Despite the fact that all NDV strains with virulent cleavage sites are highly pathogenic and fatal to birds, there is considerable variation in their MDT and ICPI values, suggesting that the F-cleavage site alone is not the sole determinant of virulence. It is widely held that other viral genes within NDV also contribute to its virulence through distinct mechanisms [56–65].

As an important structural protein, the M proteins of NDV and other paramyxoviruses mediate the whole process of viral assembly and budding, and the differences in its operational performance will inevitably affect the proliferation efficiency and virulence of the virus. In previous report, we confirmed the existence of a classical NES sequence on M protein (i.e., aa 257 to 266), which plays a very important role in the nuclear export of M protein [32, 66]. In this study, we found that the amino acid sequences of the NDV M protein NES motif conform to the standard ΦX(2–3)ΦX(2–3)ΦX pattern; however, there are differences in the genotypes at residues 257, 259, 263, and 265. The NES motif of the existing strains can be divided into six types. Among them, the LaSota-NES, Herts/33-NES, and JS10-NES are predominantly found in early-lineage NDV M proteins, while the other three motifs—Italy2736-NES, Italien-NES, and JSD0812-NES—are mainly present in recent-lineage NDV strains. Among these, the NES motifs of the LaSota strain exhibit the weakest nuclear export activity, whereas those of the JSD0812 strain demonstrate the strongest. Considering the genetic evolution of NES amino acid sites and their impact on nuclear export efficiency, we speculate that other genotypes'NES motifs are transitional types between LaSota-NES and JSD0812-NES.

Both LaSota-NES^257−266^ and Herts/33-NES^257−266^ motifs are derived from early lineage NDV strains, and the R263S mutation is the only difference between them. However, the R/S substitution at residue 263 does not have a significant effect on the reporter plasmid Rev(1.4)-GFP (Figures 2 and 3) but does influence the M protein's nuclear export efficiency (Figure 4). Our previous study has found that the R263S mutation may be related to the ubiquitination of K247 in NDV M protein and NLS-NES interaction, indirectly affecting nuclear export [23].

Genotypes VIII, VII, and the subsequent XI-XIV, XVII, and XVIII have been the predominant since the fourth and most recent pandemic recently [3, 4, 7]. The NES motifs of the M proteins in these recent-lineage strains have evolved to become ^257^IEEEKIRRLNL^266^, with the highest nuclear export capability. This primarily involves mutations at three sites: L257I, K259E, and D265N. As shown in Figure 4, the NES motif is located at the dimer interface, and all of its amino acid residues are exposed on the surface of the M protein. Mutations involving acidic and basic amino acids, such as K259E and D265N, will lead to direct changes in charge distribution within this domain, which may alter the nuclear export efficiency of the M protein. Further, other recent genotypes, such as V and VI, were associated with the second major pandemic of NDV [3, 4]. The M protein NES motifs of these strains and their derived genotypes XXI, XIX, and XX exhibit mutations in some of the four key sites of the JSD0812-NES, with a nuclear export function intermediate between the LaSota-NES and JSD0812-NES. This indicates that the NES motif of the M protein has evolved along with the NDV genotypes.

In this study, we found that the nuclear export of the NDV M protein, especially mediated by NES, is associated with the host CRM1 pathway. The leucine-rich NES is the most common and extensively studied type of nuclear export signal sequence and is utilized across all eukaryotes [67, 68]. The host nuclear export receptor, CRM1, is responsible for the recognition and interaction with NES for subsequent export [69–71]. Our analysis revealed that the M protein NES possesses a standard hydrophobic NES motif, suggesting a high probability of recognition by host CRM1. After transfection of different plasmids, the cells were treated with LMB, a highly potent and specific inhibitor of CRM1, and then detected for the impact of the CRM1 pathway on M protein nuclear export, as previously reported [72–74]. To rule out the influence of any unknown factors within the M protein that might affect nuclear export and import, only the NES^257−266^ motif was incorporated into the reporter plasmid. As anticipated, it is apparent that LMB exhibits a significantly more pronounced effect on the NES-containing reporter plasmids compared to those that express the M protein.

Our results showed that both NES-mediated and K247 ubiquitination-mediated nuclear export could be effectively inhibited, indicating the crucial role of CRM1 in the nuclear trafficking process of the M protein. K247, as a ubiquitination site in the NLS motif, can promote the nuclear export of M protein. Our results further confirmed that the promoting effect of K247 on the nuclear export of M protein is regulated by CRM1. The possible mechanism is that K247 accelerates the nucleo-plasmic shuttle process by promoting nuclear input, but the nuclear export process is still determined by the NES motif. Therefore, LMB can also affect the nuclear export of M protein at K247. In addition, it has been found that the binding affinity and nuclear export activity of NESs and CRM1 are linearly correlated with the dissociation constant (Kd) of NESs, which ranges from tens of nanomolar to tens of micromolar [75]. This report may explain the differences in the efficiency of NES motif mediated nuclear export between different genotypes (Figure 2C), and combined with our results (Figure 4) and the report above, a strong guess can be made that changes in key amino acid sites affect the binding of NES motif to CRM1, resulting in differences in the efficiency of NES motif mediated nuclear export.

**Figure 8:**
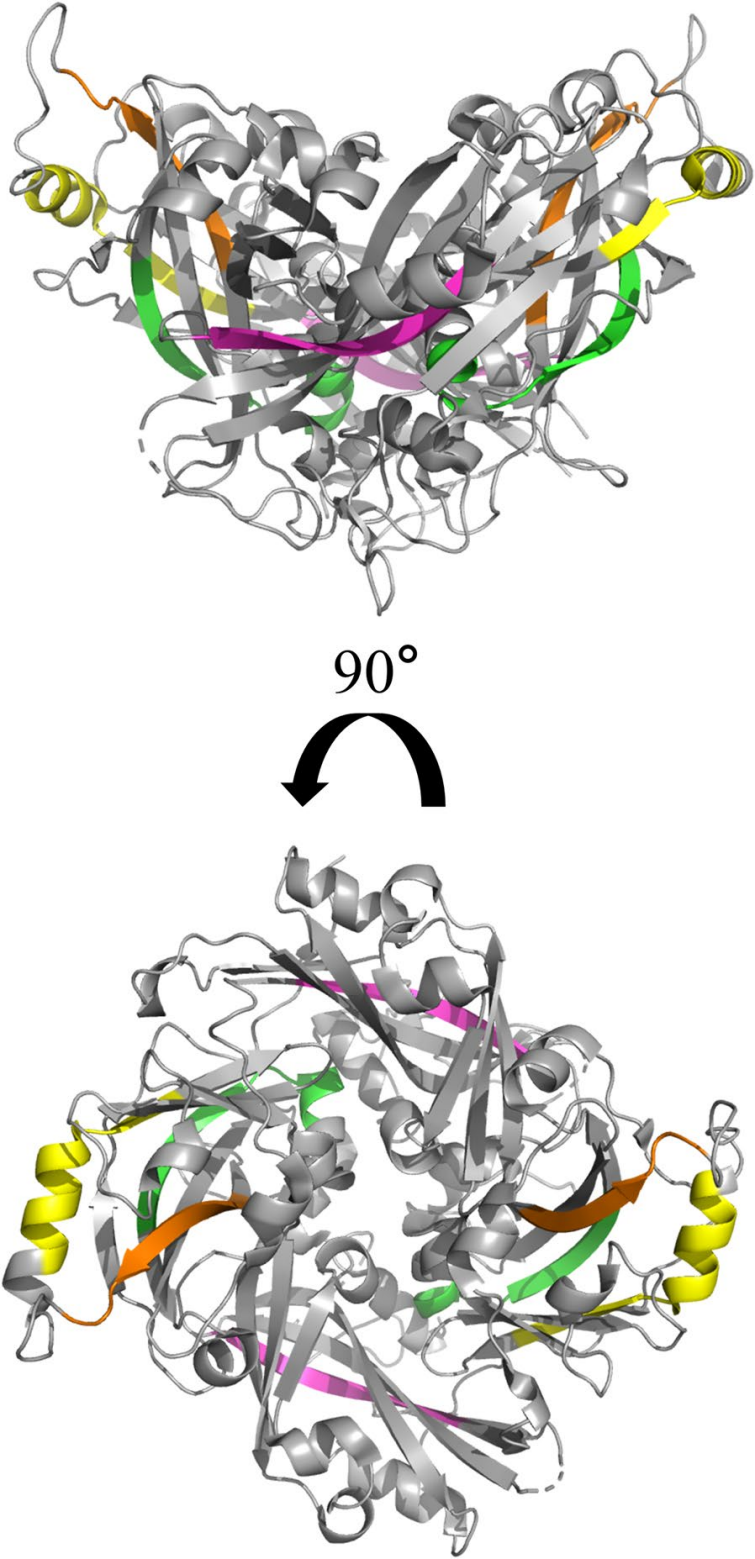
**The spatial position of four NES motifs in the M protein of NDV.** Structural model of NDV-M protein dimer generated using PyMOL (version 3.1) based on the crystal structure solved previously (PDB: 4G1G). The NES^257–266^ motif was highlighted in yellow. The other NES motifs, including NES^169–178^, NES^235–245^, and NES^314–327^ were identified in the previous report and shown in pink, orange and green, respectively.

However, our study did not cover all nuclear export pathways of M proteins. In this study, LMB treatment significantly inhibited the nuclear export of M proteins and related mutants, but did not completely confine M proteins within the nucleus, suggesting that M proteins may be exported to the nucleus via other NES sequences or other pathways. In a previous report, Duan identified three other potential NES motifs in NDV M protein (designated as NES^169−178^, NES^235−245^, and NES^314−327^) and proposed that the nuclear export of the M protein is independent of the CRM1 pathway [37], suggesting the existence of alternative nuclear export pathways. Moreover, from a structural standpoint, NES^169−178^, NES^235−245^, and NES^314−327^ are distinct from NES^257−266^ and are buried within the M protein (refer to Figure 8). This positioning could potentially reduce their interaction with CRM1. This suggests that there may be more than one NES mediating the nuclear export of M protein, and not all NES are dependent on CRM1 pathway. Similar observations have been documented in the study of influenza. In the NP protein of influenza A virus, three NES motifs were identified as responsible for nuclear export, among which the export facilitated by NES1 and NES2 is CRM1-independent, whereas the export mediated by NES3 is CRM1-dependent [75]. These results suggest that viruses may evolve multiple parallel mechanisms to maintain their life cycle in order to adapt to different environments.

Our research revealed that the efficacy of the M protein NES^257−266^ motif in facilitating the egress of M protein from the nucleus is an important factor in modulating the biological activity of NDV. Through the application of reverse genetic techniques, we have established that substituting the weak NES^257−266^ from the LaSota strain with the potent NES^257−266^ from the JSD0812 strain significantly improves the replicative efficiency of the recombinant virus in both chick embryos and cell culture systems (Table 1). Prior research has suggested that enhancing nuclear export efficiency can directly lead to increased budding efficiency, thereby boosting viral replication and enhancing pathogenicity [23]. Our findings in this study further validate this notion, showing an obvious increase in both the viral titer and virulence of the recombinant viruses. Moreover, our data from Figures 7D and E indicate that the rLaSota-QMS strain carrying the JSD0812 NES^257−266^ motif exhibits a significantly greater capacity for replication in HeLa-1 cells compared to DF1 cells. The DF1 cell line is a chicken-derived immortalized embryonic fibroblast, while the HeLa cells are a human cervical carcinoma epithelial cell line. This finding implies that NES motifs that have evolved in recent NDV strains may play a role in enhancing the replication of NDV within mammalian hosts. In other words, the evolution of these NES motifs may facilitate the virus's adaptation to its host environment. However, further investigation is needed to verify this phenomenon.

In addition, the spatial proximity of the NES and the ubiquitination site on K247 (Figure 4), along with their CRM1 dependence for nuclear export, implies a possible functional convergence between the two. The K247 and NES exhibited a synergistic enhancement effect only in certain data sets in this study, such as the growth curves of the recombinant viruses, which clearly demonstrate a pronounced synergistic interaction, suggesting that the underlying molecular mechanisms require further in-depth investigation. Nonetheless, it is certain that regardless of the approach used to enhance the nuclear export efficiency of the M protein, it can effectively increase the virus's titer and virulence.

For NDV, the F protein cleavage site is the most critical determinant of virulence. Other viral proteins, such as the V protein, the HN protein, and RNA-dependent RNA polymerase (RdRp), have also been found to influence virulence through various mechanisms [60]. In this study, our results indicate that the NES within the M protein can also affect viral virulence, although its impact is not as pronounced as that of the F protein cleavage site. As shown in Table 1, modification of the NES has indeed resulted in a significant increase in the titer and pathogenicity indices of the non-pathogenic LaSota vaccine strain, which may be attributed to the enhanced efficiency of viral replication. Correspondingly, previous studies on NP-P-L support the view that replication efficiency plays a pivotal role in viral virulence [57, 59]. By improving the performance of NP, P, and L proteins, which in turn increases the RdRp’s efficiency in synthesizing viral RNA, it is possible to accelerate viral replication and simultaneously enhance viral virulence. Although the NES has a relatively minor effect on the virulence of NDV, it is reasonable to infer that the cumulative effects of enhancements across multiple genes and motifs during the NDV evolutionary process would lead to substantial changes in the virus’s biological properties, potentially resulting in more virulent strains.

In summary, our study indicates that the NES^257−266^ motif within the NDV M protein has become more pronounced throughout the evolution of NDV, and the current predominant virulent NDV strains have developed the most effective NES motif. By modulating the nuclear export efficiency of the M protein, this NES^257−266^ motif influences both the replication and virulence of NDV, thereby acting as a determinant of its virulence.

## Supplementary Information

**Additional file 1. The GenBank accession number of NDV strains used in this study.**

**Additional file 2.** **The description of the recombinant plasmids and recombinant viruses constructed in this study.**

## Data Availability

Sequences of all primers, details of the cloning, and the plasmids used in this study are available upon request.
