# Supplementary material for: The CRM1-dependent NES257−266 motif in the matrix protein: another factor influencing Newcastle disease virus propagation and virulence
Source: Vet Res. 2025 Jul 1;56:126. doi: 10.1186/s13567-025-01552-6 (PMC12211488; doi:10.1186/s13567-025-01552-6)
Supplement: Supplementary file 1 — Additional file 1. The GenBank accession number of NDV strains used in this study. [file 13567_2025_1552_MOESM1_ESM.docx]

**Table S1** The GenBank accession number of NDV strains used in this study.

| **Strain** | **Class** | **Genotype** | **Accession Number** |
| --- | --- | --- | --- |
| DE-R49/99 | I | 1.2 | DQ097393.1 |
| NDV08-004 | I | 1.1.1 | FJ794269.1 |
| AUS/02/1334 | II | I | AY935490.1 |
| AUS-1252/98 | II | I | AY935493.1 |
| I-2 | II | I | AY935499.2 |
| N.Ireland/Ulster/67 | II | I | AY562991.1 |
| PHY-LMV42 | II | I | DQ097394.1 |
| AQI-ND026 | II | Ⅱ | DQ060053.1 |
| B1 | II | Ⅱ | NC_002617.1 |
| LaSota | II | Ⅱ | AF077761.1 |
| VG/GA | II | Ⅱ | EU289028.1 |
| JS/7/05/Ch | II | Ⅲ | FJ430159.1 |
| JS/9/05/Go | II | Ⅲ | FJ430160.1 |
| Mukteswar | II | Ⅲ | EF201805.1 |
| Italien | II | Ⅳ | EU293914.1 |
| Herts/33 | II | Ⅳ | AY741404.1 |
| US(CA)/211472/02 | II | Ⅴ | AY562987.1 |
| U.S.(FL)/44083/93 | II | Ⅴ | AY562986.1 |
| US/Largo/71 | II | Ⅴ | AY562990.1 |
| Dove/Italy/2736/00 | II | Ⅵ | AY562989.1 |
| Pigeon/IT-227/82 | II | Ⅵ | AJ880277.1 |
| US(CA)/1083/72 | II | Ⅵ | AY562988.1 |
| NA-1 | II | Ⅶ | DQ659677.1 |
| Guangxi7/02 | II | Ⅶ | DQ485229.1 |
| Guangxi9/03 | II | Ⅶ | DQ485230.1 |
| Guangxi11/03 | II | Ⅶ | DQ485231.1 |
| ZJ1 | II | Ⅶ | AF431744.3 |
| QH1a | II | Ⅷ | FJ751918.1 |
| F48E8 | II | Ⅸ | FJ436302.1 |
| FJ/1/85/Ch | II | Ⅸ | FJ436304.1 |
| JS/1/02/Du | II | Ⅸ | FJ436306.1 |
| JS/1/97/Ch | II | Ⅸ | FJ436305.1 |
| ZJ/1/86/Ch | II | Ⅸ | FJ436303.1 |
| MG/725/08 | II | ⅩⅠ | HQ266602.1 |
| Arequipa/Peru/2208463 | II | ⅩⅡ | PP788558.1 |
| Goose/CH/GD/E115 | II | ⅩⅡ | MK616244.1 |
| Zambia/Chiwoko | II | ⅩⅢ | MF409241.1 |
| NDV42/gopalpura | II | ⅩⅢ | KM056349.1 |
| Nigeria/KD/TW/03T/N45/720 | II | ⅩⅣ | KY171990.1 |
| DominicanRepublic/499-31 | II | ⅩⅥ | JX119193.1 |
| Nigeria/JN/469/N44/892 | II | ⅩⅦ | KY171992.1 |
| Nigeria/VRD08/385/N23 | II | ⅩⅦ | MN153810.1 |
| Nigeria/OOT/4/1/N69/914 | II | ⅩⅧ | MH392227.2 |
| Cormorant/Florida/41105 | II | ⅩⅨ | KC433530.1 |
| Bulgaria/Dolno Linevo/1160 | II | ⅩⅩ | KY042125.1 |
| Japan/Ibaraki/SM87 | II | ⅩⅩ | AB853928.2 |
| Pak/Bal/BC-47 | II | ⅩⅩⅠ | OQ295863.1 |
