# Supplementary material for: The CRM1-dependent NES257−266 motif in the matrix protein: another factor influencing Newcastle disease virus propagation and virulence
Source: Vet Res. 2025 Jul 1;56:126. doi: 10.1186/s13567-025-01552-6 (PMC12211488; doi:10.1186/s13567-025-01552-6)
Supplement: Supplementary file 2 — Additional file 2. The description of the recombinant plasmids and recombinant viruses constructed in this study. [file 13567_2025_1552_MOESM2_ESM.docx]

**Table S2** The description of the recombinant plasmids and recombinant viruses constructed in this study.

| **Full name** | **Plasmid/Virus** | **Abbreviation  in figures** | **Referred  figures** | **Description** |
| --- | --- | --- | --- | --- |
| Rev(1.4)-GFP | plasmid | Vector | 2 | The reporter plasmid vector for NES detection |
| pRev-GFP-Herts/33-NES | plasmid | Herts/33-NES | 2 | The Herts/33-NES^257-266^ motif was inserted into the C-terminal of the GFP gene of the reporter plasmid Rev(1.4)-GFP |
| pRev-GFP-LaSota-NES | plasmid | LaSota-NES | 2, 6 | The LaSota-NES^257-266^ motif was inserted into the C-terminal of the GFP gene of the reporter plasmid Rev(1.4)-GFP |
| pRev-GFP-Italy2736-NES | plasmid | Italy2736-NES | 2 | The Italy2736-NES^257-266^ motif was inserted into the C-terminal of the GFP gene of the reporter plasmid Rev(1.4)-GFP |
| pRev-GFP-Italien-NES | plasmid | Italien-NES | 2 | The Italien-NES^257-266^ motif was inserted into the C-terminal of the GFP gene of the reporter plasmid Rev(1.4)-GFP |
| pRev-GFP-JS10-NES | plasmid | JS10-NES | 2 | The JS10-NES^257-266^ motif was inserted into the C-terminal of the GFP gene of the reporter plasmid Rev(1.4)-GFP |
| pRev-GFP-JSD0812-NES | plasmid | JSD0812-NES | 2, 6 | The JSD0812-NES^257-266^ motif was inserted into the C-terminal of the GFP gene of the reporter plasmid Rev(1.4)-GFP |
| pRev-GFP-LaSota-NES-L257I | plasmid | L257I | 3 | An L257I mutation was introduced in the pRev-GFP-LaSota-NES |
| pRev-GFP-LaSota-NES-K259E | plasmid | K259E | 3 | An K259E mutation was introduced in the pRev-GFP-LaSota-NES |
| pRev-GFP-LaSota-NES-S263R | plasmid | S263R | 3 | An S263R mutation was introduced in the pRev-GFP-LaSota-NES |
| pRev-GFP-LaSota-NES-D265N | plasmid | D265N | 3 | An D265N mutation was introduced in the pRev-GFP-LaSota-NES |
| pRev-GFP-JSD0812-NES-I257L | plasmid | I257L | 3 | An I257L mutation was introduced in the pRev-GFP-JSD0812-NES |
| pRev-GFP-JSD0812-NES-E259K | plasmid | E259K | 3 | An E259K mutation was introduced in the pRev-GFP-JSD0812-NES |
| pRev-GFP-JSD0812-NES-R263S | plasmid | R263S | 3 | An R263S mutation was introduced in the pRev-GFP-JSD0812-NES |
| pRev-GFP-JSD0812-NES-N265D | plasmid | N265D | 3 | An N265D mutation was introduced in the pRev-GFP-JSD0812-NES |
| pRev-GFP-Italy2736-NES-G259K | plasmid | G259K | 3 | An G259K mutation was introduced in the pRev-GFP-Italy2736-NES |
| pCMV-14-LaSota-M-3×FLAG | plasmid | LaSota-M | 4, 5, 6 | The eukaryotic expression plasmid expressing the LaSota M protein fused with a 3×flag tag |
| pCMV-14-LaSota-M-L257I-3×FLAG | plasmid | LaSota-M-L257I | 4 | An L257I mutation was introduced in pCMV-14-LaSota-M-3×FLAG |
| pCMV-14-LaSota-M-K259G-3×FLAG | plasmid | LaSota-M-K259G | 4 | An K259G mutation was introduced in pCMV-14-LaSota-M-3×FLAG |
| pCMV-14-LaSota-M-K259E-3×FLAG | plasmid | LaSota-M-K259E | 4 | An K259E mutation was introduced in pCMV-14-LaSota-M-3×FLAG |
| pCMV-14-LaSota-M-S263R-3×FLAG | plasmid | LaSota-M-S263R | 4 | An S263R mutation was introduced in pCMV-14-LaSota-M-3×FLAG |
| pCMV-14-LaSota-M-D265N-3×FLAG | plasmid | LaSota-M-D265N | 4 | An D265N mutation was introduced in pCMV-14-LaSota-M-3×FLAG |
| pCMV-14-LaSota-M-R247K-3×FLAG | plasmid | LaSota-M-R247K | 5, 6 | An R247K mutation was introduced in pCMV-14-LaSota-M-3×FLAG |
| pCMV-14-LaSota-M-JSD0812-NES-3×FLAG | plasmid | LaSota-M-JSD0812-NES | 5, 6 | The LaSota-NES^257-266^ motif was replaced by the JSD0812-NES^257-266^ motif in pCMV-14-LaSota-M-3×FLAG |
| pCMV-14-LaSota-M-R247K-JSD0812-NES-3×FLAG | plasmid | LaSota-M-R247K-JSD0812-NES | 5, 6 | An R247K mutation was introduced in pCMV-14-LaSota-M-JSD0812-NES-3×FLAG |
| pCMV-14-JSD0812-M-3×FLAG | plasmid | JSD0812-M | 5, 6 | The eukaryotic expression plasmid expressing the JSD0812 M protein fused with a 3×flag tag |
| pCMV-14-JSD0812-M-K247R-3×FLAG | plasmid | JSD0812-M-K247R | 5 | An K247R mutation was introduced in pCMV-14- JSD0812-M-3×FLAG |
| pCMV-14-JSD0812-M-LaSota-NES-3×FLAG | plasmid | JSD0812-M-LaSota-NES | 5 | The JSD0812-NES^257-266^ motif was replaced by the LaSota-NES^257-266^ motif in pCMV-14-JSD0812-M-3×FLAG |
| pCMV-14-JSD0812-M-K247R-LaSota-NES-3×FLAG | plasmid | JSD0812-M-K247R-LaSota-NES | 5 | An K247R mutation was introduced in pCMV-14-JSD0812-M-LaSota-NES-3×FLAG |
| rLaSota-wt | virus | rLaSota-R247K | 7 | A recombinant LaSota strain without any mutation rescued from the full-length genomic clone via reverse genetics. |
| rLaSota-M-R247K | virus | rLaSota-R247K | 7 | A rescued recombinant LaSota strain with a R247K mutation in its M protein. |
| rLaSota-M-L257I&K259E&S263R&D265N-QMS | virus | rLaSota-QMS | 7 | A rescued recombinant LaSota strain, in which the NES^257-266^ motif of M protein was replaced by JSD0812 NES^257-266^ motif. |
| rLaSota-M-R247K&L257I&K259E&S263R&D265N-FM | virus | rLaSota-FM | 7 | A rescued recombinant LaSota strain, in which the NES^257-266^ motif of M protein was replaced by JSD0812 NES^257-266^ motif, as well as a R247K mutation was introduced. |
